# Does patient age influence procedural management of kidney trauma?

**DOI:** 10.1186/s12894-025-01879-4

**Published:** 2025-08-21

**Authors:** Nizar Hakam, Behzad Abbasi, Umar Ghaffar, Kevin D. Li, Hiren Patel, Charles P. Jones, Joseph Cuschieri, Benjamin N. Breyer

**Affiliations:** 1 Department of Urology, University of California San Francisco, San Francisco, CA USA; 2 Department of Urology, The Ohio State University Wexner Medical Center, Columbus, OH USA; 3 Division of Urologic Surgery, Departmentof Surgery, Washington University St Louis, St Louis, MO USA; 4 Department of Surgery, University of California San Francisco, San Francisco, CA USA; 5 Department of Epidemiology and Biostatistics, University of California San Francisco, San Francisco, CA USA

**Keywords:** Renal trauma, Nephrectomy, Operative management

## Abstract

**Objectives:**

We aimed to examine the association between patient age and procedural intervention, especially nephrectomy, in patients with renal trauma in the National Trauma Data Bank (NTDB).

**Materials and methods:**

We queried the 2013–2020 NTDB for adult renal trauma patients with an American Association for the Surgery of Trauma (AAST) grade. Patients without AAST grade or with no sign of life were excluded. We constructed a multinomial logistic regression model to demonstrate the association between age and procedural interventions (renal angioembolization, renorrhaphy and nephrectomy). Models were adjusted for patient, hospital, and clinical factors.

**Results:**

Our cohort was comprised of 49,884 patients with renal trauma aged 18–89 years, of which 691 (1.4%), 995 (1.9%), and 3,366 (6.8%) underwent angioembolization, renorrhaphy, and nephrectomy, respectively. After adjusting for relevant variables, the risks of nephrectomy and angioembolization were positively associated with patient age, particularly in those 40 years of age and older. Adjusted risk of nephrectomy (OR 0.07 per 10 years of age, 95% CI 0.03–0.11, *p* < 0.001) and angioembolization (OR 0.19 per 10 years of age, 95% CI 0.14–0.24, *p* < 0.001) ranged between ≈ 0.6% and ≈ 1%, and between ≈ 0.3% and ≈ 1% across the patient age range, respectively. Plots depicting marginal effect of age is demonstrated that in patients above 35–40 years of age, a 1-year increase in age is associated with a progressively higher increase in risk of both nephrectomy and angioembolization. The adjusted risk of Renorrhaphy (OR -0.003 per 10 years of age, 95% CI -0.06-0.06, *p* = 0.92) did not vary substantially with age and the marginal effect of age was negligible across all ages.

**Conclusions:**

Older patients with renal trauma are more likely to receive procedural intervention namely nephrectomy and renal angioembolization. These results suggest possible age-related cognitive bias in renal trauma management.

**Supplementary Information:**

The online version contains supplementary material available at 10.1186/s12894-025-01879-4.

## Introduction

The kidney is a commonly injured organ in trauma and is a significant source of morbidity and mortality [1–3]. Despite the paradigm shift in renal trauma guidelines away from exploration and intervention in the past two decades, nephrectomy remains common especially in high grade renal trauma [4–6]. Whether mortality increases after undergoing a trauma nephrectomy is unclear, with some studies purporting an association [7, 8] and others not [9]. Regardless of the causal association between nephrectomy and mortality, the ultimate management goal is to avoid nephrectomy in a surviving patient.

Several studies have assessed factors associated with operative management or nephrectomy of a traumatized kidney [4, 10, 11]. Identifying modifiable risk factors is of particular importance since it can lead to further decrease in avoidable nephrectomy. As these studies focused on the role of hemodynamic and injury characteristics, patient age has not been specifically addressed. Age can be a source for cognitive bias during patient management in many conditions, one example being the left-digit bias phenomenon [12–14]. 

The aim of this study is to examine the association between patient age and procedural intervention, especially nephrectomy, in patients with renal trauma in the National Trauma Data Bank (NTDB). Our hypothesis is that an independent association between age and nephrectomy is likely related to heterogenous threshold of performing nephrectomy in different age groups. Awareness of such discrepancies may lead to fewer nephrectomies in older patients.

## Materials and methods

### Study population

De-identified patient data was collected from the 2013 to 2020 NTDB, a hospital-based trauma registry with comprehensive patient, injury severity, and management data [15]. Adult (age ≥ 18 years) renal trauma patients with American Association for the Surgery of Trauma (AAST) kidney injury grades were identified using Abbreviated Injury Scale (AIS) codes (AIS 541612 - AAST grade I; AIS 541622 - AAST grade II; AIS 541624 - AAST grade III; AIS 541626 - AAST grade IV; AIS 541628 - AAST grade V) [16]. Patients who arrived with no signs of life were not included. Institutional Review Board exemption was provided since data were deidentified. Guidelines of the STROBE statement for observational studies were followed [17]. 

### Study endpoint

The primary endpoint was whether a patient underwent renal angioembolization, renorrhaphy, or nephrectomy. An indicator for use of renal angioembolization was available in the data. International Classification of Disease (ICD) codes were used to identify occurrence of renorrhaphy (ICD-9 55.81; 55.86–55.89 or ICD-10 0TQ00ZZ; 0TQ03ZZ; 0TQ04ZZ; 0TQ07ZZ; 0TQ08ZZ, 0TQ10ZZ; 0TQ13ZZ; 0TQ14ZZ; 0TQ17ZZ; 0TQ18ZZ; 0TQ30ZZ; 0TQ33ZZ; 0TQ34ZZ; 0TQ37ZZ; 0TQ38ZZ; 0TQ40ZZ; 0TQ43ZZ; 0TQ44ZZ; 0TQ47ZZ; 0TQ48ZZ) and nephrectomy (ICD-9 55.51–55.54 or ICD-10 0TT00ZZ; 0TT04ZZ; 0TT10ZZ; 0TT14ZZ; 0TT20ZZ; 0TT24ZZ; 0TT30ZZ; 0TT40ZZ; 0TB10ZZ; 0TB00ZZ; 1PC89LB).

### Statistical analysis

To understand differences in trauma profiles across age, we grouped patients in 10-year increments of age and compared their clinical characteristics. Statistical tests used included chi-square test for categorical variables, ANOVA and Kruskal-Wallis tests for normally distributed and non-normally distributed continuous variables, respectively. Multivariable analysis was conducted using multinomial logistic regression with outcome levels including angioembolization, renorrhaphy, and nephrectomy compared to a reference group of no intervention. Patient age was the primary predictor of interest. The models were adjusted for socioeconomic factors (sex, race, trauma center level), injury specific factors (renal injury grade, penetrating mechanism, injury severity scale (ISS)), hemodynamic parameters (transfusion in the first 4 h, systolic blood pressure, pulse rate), and chronic renal failure (defined as preexisting renal failure requiring periodic peritoneal dialysis, hemodialysis, hemofiltration, or hemodiafiltration). We also adjusted for whether the patient underwent liver, spleen, or bowel surgery which have been previously shown to be associated with nephrectomy [4]. We used the modified 5-item frailty index (mFI-5) to quantify and adjust for patient frailty, which is a validated and commonly used metric repeatedly shown to correlate with clinical outcomes in trauma [18–24]. The mFI-5 can be calculated using comorbidities consistently recorded in the NTDB: chronic obstructive pulmonary disease, congestive heart failure, diabetes mellitus, functionally dependent health status, and hypertension. One point is assigned for each present comorbidity and thus the mFI-5 ranges between 0 and 5. All continuous variables were modeled with cubic splines to allow for a non-linear association. Plots demonstrating the regression predicted probability of outcome and marginal effect of age were constructed to demonstrate the association between age and nephrectomy. Since clinical practices for acute renal trauma management varied over time, we performed a sensitivity analysis where year of surgery was included as an independent factor in the adjusted models. No differences were observed between the models with and without year of surgery. All statistical analysis was performed using Stata^®^ version 17.

### Sensitivity analysis

We performed multiple sensitivity analyses. We repeated our analysis in subgroups of renal injury grade with minimal changes to the findings. Furthermore, some patients could have had a kidney trauma-related intervention after initial non-operative management, which is not directly captured in NTDB. Thus we performed a sensitivity analysis where we repeated the analysis with redefined primary endpoints to only include patients that had renal angioembolization / renorrhaphy / nephrectomy combined with an “Operating Room” Emergency Department disposition. Otherwise we assumed that there was a clinical decision for initial non-operative management for that patient.

## Results

Our cohort was comprised of 49,884 patients with renal trauma (Table 1). Mean age was 39.6 years (SD 17.8, range 18–89). Most patients were males (75.2%), had blunt trauma (81.6%) and had a grade III renal trauma or higher (55.2%). Nearly one-quarter received a transfusion within the first 4 h. Compared to younger age groups, older patients had lower proportions of high grade renal trauma, fewer penetrating injuries, and received more transfusions within the first 4 h (Table 2). Older age groups also had progressively higher SBP, lower pulse rate and ISS, and higher mFI-5 scores (Table 2).

**Table 1:** Description of study population

	No.	%
Age mean (SD)	39.6 (17.8)	
Male sex	37,516	75.2
Race		
White	31,542	63.2
Black	10,449	20.9
Asian	898	1.8
Other / Unknown	6995	14
Renal trauma grade		
I	10,450	20.9
II	11,880	23.8
III	14,965	30
IV	9074	18.2
V	3515	7.1
Penetrating mechanism	9189	18.4
SBP mean (SD)	124.3 (29.2)	
Pulse mean (SD)	95.6 (24.5)	
ISS median (IQR)	21 (14–29)	
Transfusion (4 h)	12,413	24.9
Trauma center level		
I	24,651	49.4
II	10,564	21.2
III	1238	2.5
Unknown	13,431	26.9
Angioembolization	691	1.4
Renorrhaphy	995	1.9
Nephrectomy	3366	6.8
Liver surgery	12,484	25
Spleen surgery	15,184	30.4
Bowel surgery	16,549	33.2
Chronic renal failure	290	0.58

(SD = standard deviation; SBP = systolic blood pressure; ISS = injury severity scale; IQR = interquartile range)

**Table 2:** Injury and clinical status characteristics stratified by age group

Age group	18–29	30–39	40–49	50–59	60–69	70–79	80–89	*p* value
N	19,687	9399	6410	6015	4477	2583	1313	
HGRT^1^% (III-V)	62.6	59.3	51.3	47.4	43.3	41.5	39.6	< 0.01
HGRT % (IV-V)	29.3	27.3	22.7	20.8	17.8	19.2	19	< 0.01
Penetrating mechanism %	24.5	25.3	16.8	9.9	4.7	2.6	2.1	< 0.01
SBP^2^ mean (SD)	122.4 (26.9)	121.7 (28.2)	123.4 (30.1)	125.7 (31.2)	129.5 (32.5)	132.2 (31.7)	135.1 (32.3)	< 0.01
Pulse mean (SD)	99.6 (25.6)	98.1 (24.6)	94.9 (23.4)	91.5 (22.4)	88.4 (21.4)	84.9 (20.6)	83.4 (19.2)	< 0.01
ISS^3^ median (IQR)	22 (14–34)	22 (14–34)	21 (14–29)	20 (13–29)	18 (12–29)	17 (9–25)	14 (9–24)	< 0.01
mFI-5^4^ score								< 0.01
0 (%)	94.7	88.7	75.4	61.6	44.2	30.9	24.8	
1 (%)	4.2	8.6	17.5	25.1	33.3	39.8	43.7	
≥ 2 (%)	1.1	2.7	7.1	13.3	22.5	29.2	31.5	
Transfusion 4 h %	26	28.2	25.1	23.5	21.3	17.3	16.7	< 0.01
Angioembolization %	1.2	1.3	1.2	1.3	2	2.6	2.1	< 0.01
Renorrhaphy %	2.7	2.4	1.8	1.3	0.8	0.3	0.2	< 0.01
Nephrectomy %	8.2	8.4	6.3	4.6	3.7	3.2	2.9	< 0.01

^1^HGRT = high grade renal trauma

^2^SBP = systolic blood pressure

^3^ISS = injury severity scale

^4^mFI-5 = 5-component modified frailty index

**Fig. 1:**
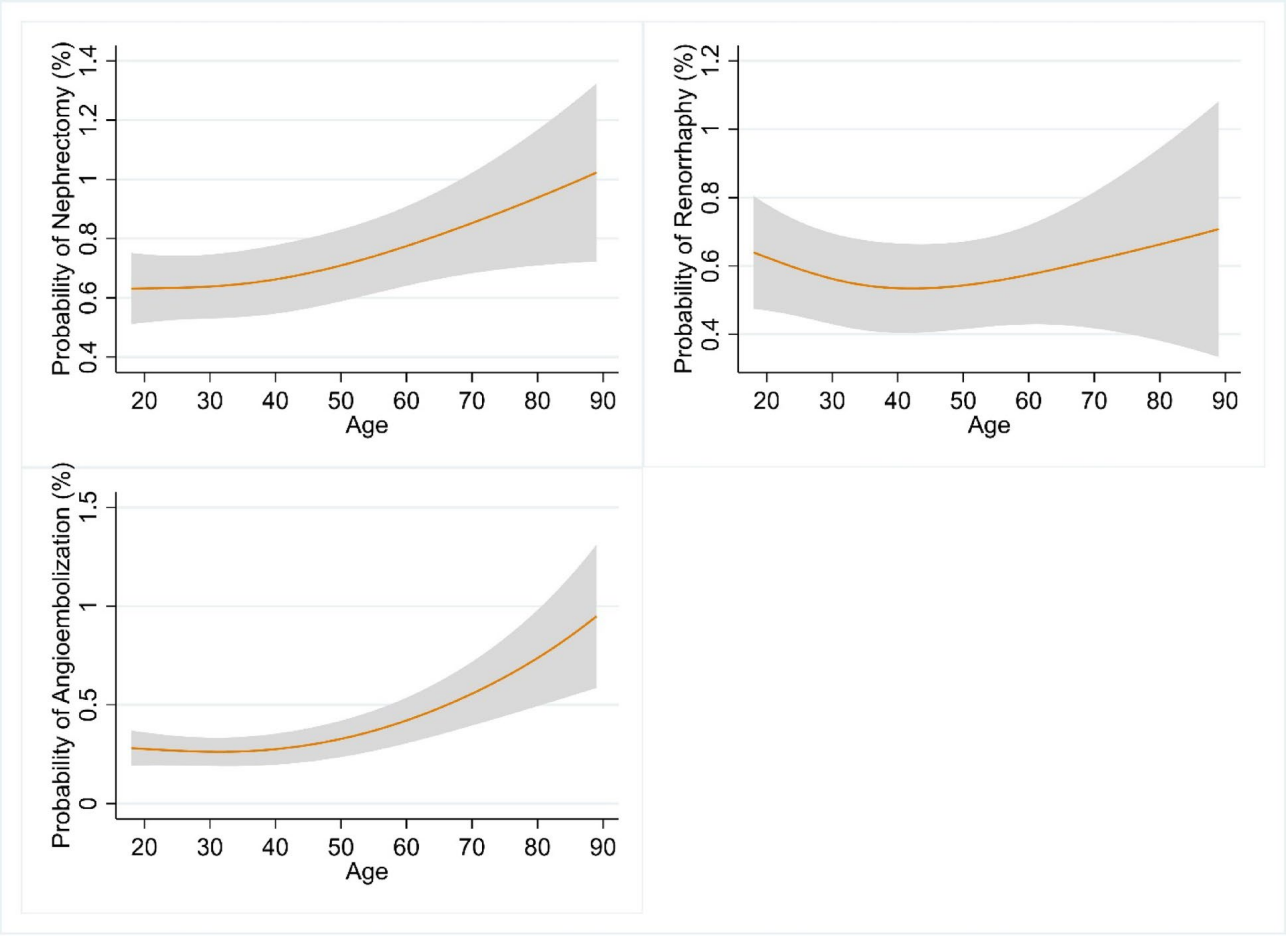
Logistic regression model predicted probability of nephrectomy, renorrhaphy, and angioembolization as a function of age (cubic spline fit). Caption: Models adjusted for patient frailty (mFI-5 score), sex, race, trauma center level, renal injury grade, transfusion in first 4 h, penetrating mechanism, systolic blood pressure, pulse rate, injury severity scale, concurrent bowel, liver, spleen surgery, chronic renal failure. Shaded area represents 95% confidence interval

**Fig. 2:**
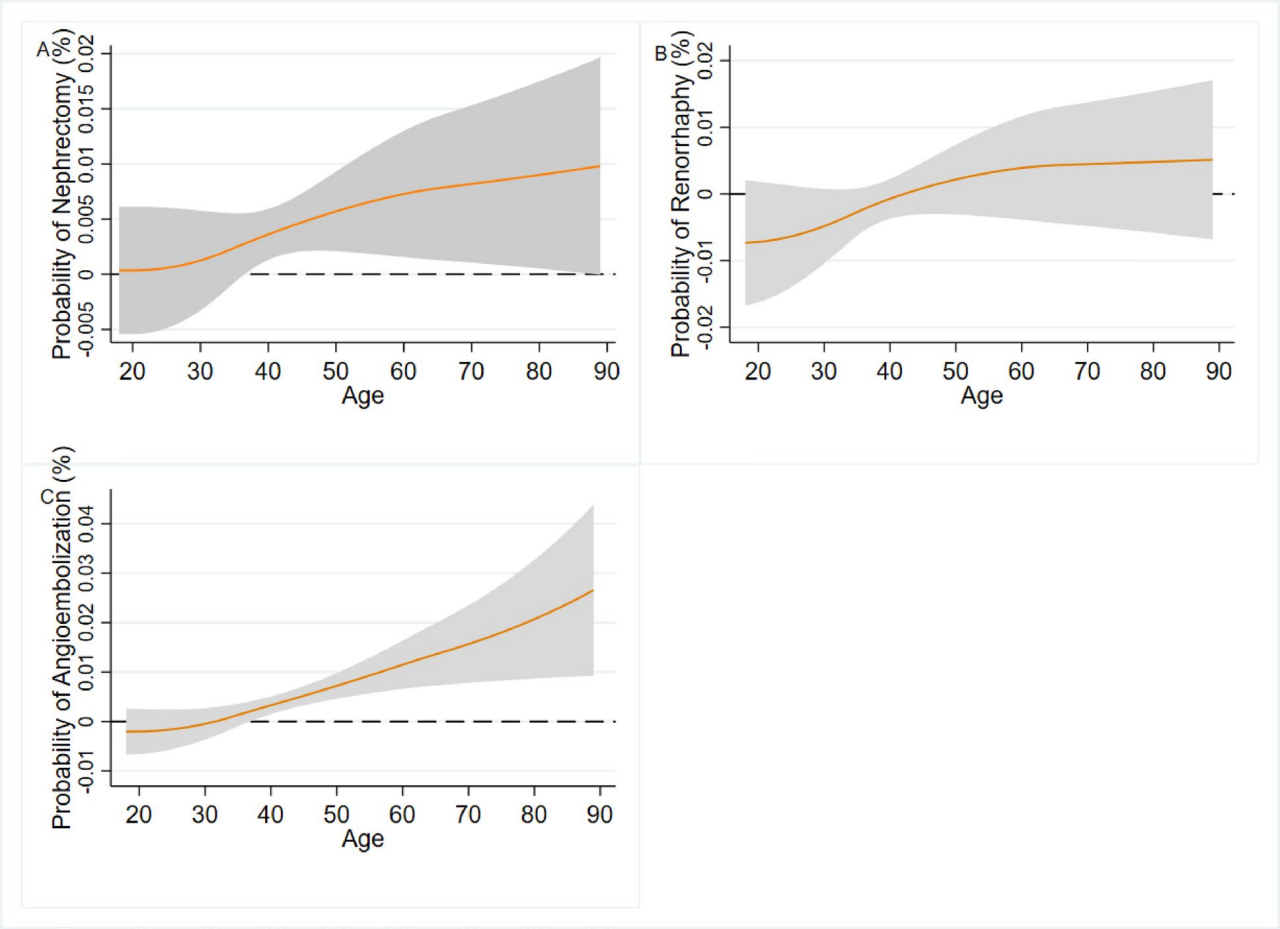
Marginal effect of age demonstrating the change in predicted probability of nephrectomy (A), renorrhaphy(B), and angioembolization (C) for a 1-year change in patient age. Caption: Models were adjusted for patient frailty (mFI-5 score), sex, race, trauma center level, renal injury grade, transfusion in first 4 h, penetrating mechanism, systolic blood pressure, pulse rate, injury severity scale, concurrent bowel, liver, or spleen surgery, chronic renal failure. Shaded area represents 95% confidence interval

Rates of procedural intervention were as follows: angioembolization *n* = 691 (1.4%), renorrhaphy *n* = 995 (1.9%), nephrectomy *n* = 3,366 (6.8%). After adjusting for relevant variables, higher age was significantly associated with increased nephrectomy (OR 0.07 per 10 years of age, 95% CI 0.03–0.11, *p* < 0.001) and angioembolization (OR 0.19 per 10 years of age, 95% CI 0.14–0.24, *p* < 0.001). The probabilities of nephrectomy and angioembolization across age are demonstrated in Fig. 1, and showed a positive association especially in those 40 years of age and older. Adjusted risk of angioembolization increased from ≈ 0.3% to ≈ 1%, and that of nephrectomy increased from ≈ 0.6% to ≈ 1% between the youngest and oldest patients. Model-derived marginal effect of age is depicted in Fig. 2 and demonstrates that in patients above 35–40 years of age, a 1-year increase in age is associated with a progressively higher increase in risk of both angioembolization and nephrectomy. For example, at age 50 a 1-unit increase in age is associated with ≈ 0.005% increase in nephrectomy (1 more in 20000 patients). At age 60 a 1-unit increase is associated with ≈ 0.0075% increase in nephrectomy (1 more in 13333 patients). The adjusted risk of renorrhaphy (Fig. 1) did not seem to vary substantially with age and the marginal effect of age was negligible across all ages (Fig. 2). We did not observe an association between patient frailty and odds of any of the interventions (Table 3).

**Table 3:** Logistic regression model adjusted odds ratios (OR) for the association between patient frailty (mFI-5 score) and intervention with angioembolization, renorrhaphy, and nephrectomy

mFI-5	OR for Angioembolization	95% CI	*p* value
0	Reference		
1	0.99	0.77–1.29	0.99
≥ 2	1.23	0.92–1.65	0.17

	OR for Nephrorrhaphy	95% CI	p value
0	Reference		
1	1.11	0.88–1.41	0.38
≥ 2	1.32	0.93–1.88	0.12

	OR for Nephrectomy	95% CI	p value
0	Reference		
1	0.93	0.79–1.09	0.39
≥ 2	1.04	0.82–1.31	0.76

Models adjusted for patient age, sex, race, trauma center level, renal injury grade, transfusion in first 4 h, penetrating mechanism, systolic blood pressure, pulse rate, injury severity scale, concurrent bowel, liver, spleen surgery, chronic renal failure

### Sensitivity analysis

There were 245 (0.5%) patients who received renal angioembolization, 893 (1.8%) who received renorrhaphy, and 2979 (5.9%) who received nephrectomy with an “Operating Room” Emergency Department disposition. Results of sensitivity analysis are presented in Supplementary Figs. 1 and 2 and were not substantially different than the main analysis.

## Discussion

In a population of nearly 50,000 adult patients with renal trauma, we found renal angioembolization and nephrectomy more likely to occur with increasing patient age after adjusting for injury severity and patient clinical characteristics. The increased intervention rate is most prominent in elderly patients. The notion that higher utilization of nephrectomy did not coincide with less use of surgical repair or angioembolization indicates possible overtreatment of older patients with nephrectomy, as younger patients with comparable renal injuries were managed less aggressively. This study is the first to demonstrate this association and presents a novel risk factor in addition to known trauma nephrectomy risk factors: unstable hemodynamic status, higher injury grade, and penetrating mechanism [4, 6, 11]. 

Our findings may exemplify age-related cognitive bias in patient management. Several studies have addressed different aspects of age bias in the clinical context, most commonly left-digit bias in which the left-most digit of a number affects decision making. Brant et al. examined management trends for localized prostate cancer in national registries and reported that patients 69 years of age were significantly more likely to receive recommendation for prostatectomy and less likely to get radiation therapy, compared to 70-year-old patient population [12]. In another study of the National Cancer Database, left-digit bias was demonstrated in a similar fashion, where potentially curable grade I-III rectal cancer patients had higher odds of receiving guideline-adherent therapy if they were 58–59 or 78–79 years old compared to patients aged 60 or 80, respectively [14]. Further, similar to our results, their analysis revealed that the chances of guideline-adherent therapy decreased progressively with every decade advancement of age [14]. 

Conservative management of traumatized kidneys enables nephron preservation, although the impact of nephrectomy on patient morbidity and mortality is poorly evidenced and subject of debate. In a recent study of over 40,000 patients in the NTDB, patients undergoing nephrectomy between 2007 and 2016 were found to be at 80% higher odds of inpatient mortality compared to none-nephrectomy patients [7]. Other studies have highlighted the role of nephrectomy in sepsis and multi-organ dysfunction and subsequent mortality [8, 25]. On the other hand, McCormick et al. examined the Trauma Quality Improvement Program database and performed a propensity score matched analysis based on wide demographic, co-morbidity, and physiologic characteristics and could not detect an association between nephrectomy and mortality [9]. Nevertheless, the desire to avoid unnecessary nephrectomy is intuitive, and our results suggest there might be room for further decreasing nephrectomy rates in older patients.

Nonoperative management of solid organ injuries has gained traction in recent years as it allows for organ preservation. Advanced age is no longer considered a mandatory indication for operative management in spleen and liver trauma [26]. The predictive role for age for the success or failure of nonoperative management of kidney injury is scarce. A study of 206 cases with grade IV and V blunt renal injuries found that patients older than 55 years had 6-fold higher odds of nonoperative approach failure compared to younger patients [27]. In another study of 117 patients with high grade renal trauma, increasing age was reportedly associated with higher failure of conservative management failure [28]. Despite identifiable flaws in both those analyses such as dichotomizing age using a cut-off point and limited adjustment for confounders without a priori selection, they may lead to trauma surgeons’ reluctance to implement conservative management for older patients in fear of poor outcomes. It should be mentioned that none of the current trauma guidelines endorse the consideration of a patient’s advanced age as a driving factor for management decision.

The main limitation to this study is residual confounding from unmeasured or even unmeasurable factors. Inaccuracies in data entry or data coding may exist as with any large database. Moreover, the data does not include details about the providers making clinical decisions (such as trauma surgeons vs. urologists) or intent for treatment (planned surgery vs. surgery after trial of non-operative management) which could lead to unmeasured confounding. Finally, NTDB is dependent on the voluntary participation of trauma centers and is not necessarily nationally representative.

## Conclusions

Older patients with renal trauma are more likely to receive procedural intervention, namely renal angioembolization and nephrectomy. These results suggest age-related cognitive bias in renal trauma management and underscore the need for proper clinical judgement by renal trauma providers to unnecessary nephrectomy where unnecessary. We advise careful decision making while managing renal injuries especially in elderly patients with frail cardiovascular systems who have lower reserve to tolerate substantial blood loss. Further studies demonstrating the impact of increased procedural intervention in older populations or reduced intervention in younger populations are warranted.

## Electronic supplementary material

Below is the link to the electronic supplementary material.

Supplementary Material 1: Figure 1. Logistic regression model predicted probability of nephrectomy, renorrhaphy, and angioembolization in patients with an “Operating Room” Emergency Department disposition as a function of age (cubic spline fit).

Supplementary Material 2: Figure 2. Marginal effect of age demonstrating the change in predicted probability of nephrectomy (A), renorrhaphy(B), and angioembolization (C) in patients with an “Operating Room” Emergency Department disposition for a 1-year change in patient age.

## Data Availability

The data that support the findings of this study are available from the American College Surgeons (https://www.facs.org/quality-programs/trauma/quality/national-trauma-data-bank/about-ntdb/) but restrictions apply to the availability of these data, which were used under license for the current study, and so are not publicly available. Data are not available from the authors without permission of the American College of Surgeons.

## Abbreviations

AAST: American association for surgery of trauma
AIS: abbreviated injury scale
NTDB: national trauma databank
ICD: international classification of diseases
ISS: injury severity scale
