# Supplementary figures and images for: Does patient age influence procedural management of kidney trauma?
Source: BMC Urol. 2025 Aug 21;25:208. doi: 10.1186/s12894-025-01879-4 (PMC12369115; doi:10.1186/s12894-025-01879-4)

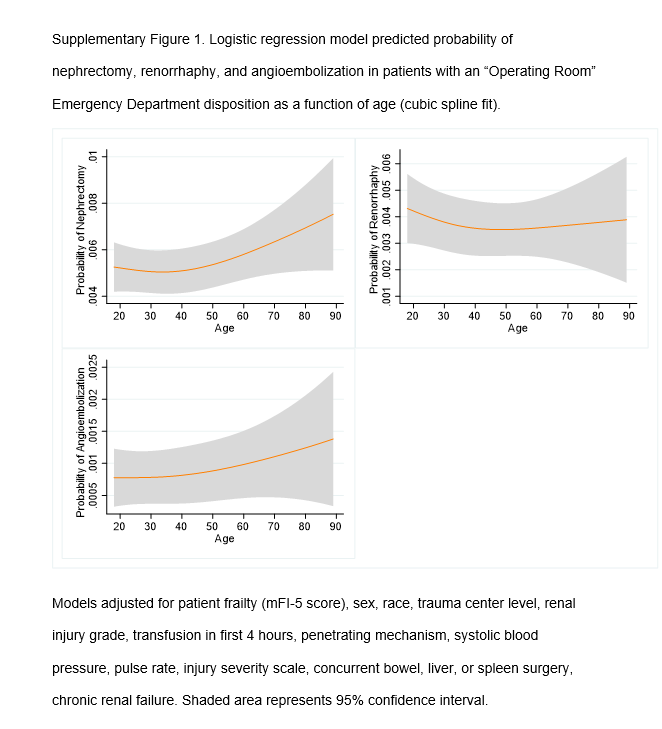

Supplement: Supplementary file 1 — Supplementary Material 1: Figure 1. Logistic regression model predicted probability of nephrectomy, renorrhaphy, and angioembolization in patients with an “Operating Room” Emergency Department disposition as a function of age (cubic spline fit). [file 12894_2025_1879_MOESM1_ESM.png]

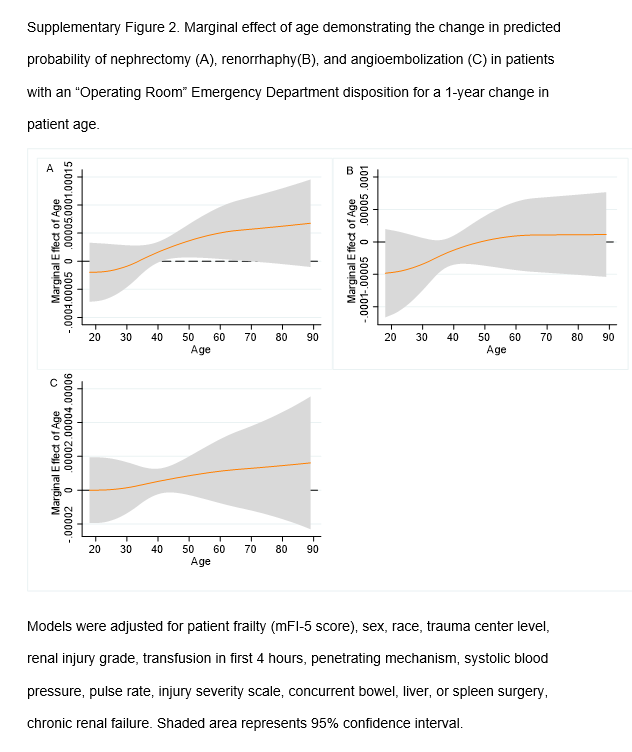

Supplement: Supplementary file 2 — Supplementary Material 2: Figure 2. Marginal effect of age demonstrating the change in predicted probability of nephrectomy (A), renorrhaphy(B), and angioembolization (C) in patients with an “Operating Room” Emergency Department disposition for a 1-year change in patient age. [file 12894_2025_1879_MOESM2_ESM.png]
